# How Can We Diagnose Ocular Surface Squamous Neoplasia With Optical Coherence Tomography?

**DOI:** 10.7759/cureus.36320

**Published:** 2023-03-17

**Authors:** Ceyda Başkan, Aydan Kılıcarslan

**Affiliations:** 1 Ophthalmology, Ankara Bilkent City Hospital, Ankara, TUR; 2 Pathology, Ankara Bilkent City Hospital, Ankara, TUR

**Keywords:** anterior segment, epithelial thickness, pterygium, ocular surface squamous neoplasia, optical coherence tomography

## Abstract

Aim: We aimed to evaluate the effectiveness of optical coherence tomography (OCT) in the differential diagnosis of anterior segment diseases such as ocular surface squamous neoplasia (OSSN) and pterygium.

Methods: Patients who were pre-diagnosed with either OSSN (21) or pterygium (19) between January 2020 and November 2022 were included in this retrospective study. Anterior segment photographs and anterior segment optical coherence tomography (AS-OCT) measurements were obtained from each patient. Excisional or incisional biopsy materials underwent pathological evaluation.

Results: Preoperative AS-OCT images of the patients with OSSN showed similarities with histopathological specimens. Both ocular and pathological specimens appeared to have a thicker epithelial layer with a distinct change from healthy to neoplastic epithelium. Preoperative AS-OCT images of individuals with pterygium were also comparable with histopathological samples. Both pathological and AS-OCT images of the pterygium patients showed a normal thickness epithelium and a thickened subepithelial layer under the epithelium. The mean epithelial thickness measured with AS-OCT in OSSN patients was found to be 295.3 ± 111.3 µm, while it was 80.7 ± 43.4 µm in pterygium patients. The difference was statistically significant (P<0.001). The receiver operating characteristic (ROC) curve analysis revealed a cut-off value of 97 µm for the differential diagnosis of OSSN from pterygium, with a sensitivity of 100% and specificity of 94.7%.

Conclusions: AS-OCT can be used as a noninvasive diagnostic tool for the evaluation of ocular surface lesions. Its ability to distinguish between OSSN and pterygium is demonstrated by the statistically significant difference in epithelial thickness and the significant morphological association with histopathological findings.

## Introduction

Formulating an appropriate diagnosis of ocular surface lesions is critically important. The conjunctiva, cornea, or both surfaces of the eye can be affected by ocular surface squamous neoplasm (OSSN). Squamous cell carcinoma of the conjunctiva and conjunctival intraepithelial neoplasia (CIN) are recognized as part of the range of OSSN. We can pre-diagnose OSSN clinically by looking for raised, gelatinous, papilliform, or leukoplakic lesions that frequently have feeding vessels and exhibit positive rose bengal staining. The differential diagnosis of OSSN includes pterygium, dyskeratosis, amelanotic melanoma, papilloma, scar tissue, and ocular pannus [1]. Many of these lesions are benign, although some may carry significant morbidity and mortality. Clinical examination and a detailed medical history are important for an accurate diagnosis, but in some cases, a more detailed examination is required. Ancillary tests are necessary for the detailed evaluation of the patient. Excisional or incisional biopsy is used to reach a definitive diagnosis. In some circumstances, an incisional biopsy may be adequate, but the entire lesion should always be removed when malignancy is suspected. Although biopsy is an invasive diagnostic method, it may not detect lesions that are present in the tissue that was removed [2]. New diagnostic techniques such as impression cytology and confocal microscopy are widely used, but they have some limitations. Confocal microscopy does not provide a cross-sectional view to identify the vertical and horizontal expanse of the lesion, whereas impression cytology only assesses the superficial cell layers [3,4].

Anterior segment optical coherence tomography (AS-OCT) noninvasively provides the evaluation of the conjunctiva and cornea with high axial tissue resolution and allows examining the morphological and histological features of tissues in vivo [5]. Thus, AS-OCT became a critical element in diagnosing and differentiating OSSN, particularly in the setting of concomitant ocular surface diseases. There have been several studies previously published, but these studies lack a significant number of patients, particularly cases of pterygium [5,6]. This research aimed to further assess the utility of AS-OCT as a complementary diagnostic instrument for OSSN and pterygium, a benign disease.

## Materials and methods

After receiving clearance from the hospital's ethical committee, the study was carried out at the ophthalmology department of the education and research hospital. The study was conducted in accordance with the Declaration of Helsinki and approved by the Institutional Ethics Committee of Ankara Bilkent City Hospital (IRB No. E1-22-3043). Thirty-nine patients with pre-diagnoses of anterior segment lesions such as OSSN or pterygium, whose diagnoses were confirmed histopathologically between January 2020 and November 2022 were included in the study. Patients with systemic diseases and previous eye surgery were not included in the study. Detailed anterior and posterior segment biomicroscopic evaluations were performed. All patients were scanned with the noncontact Topcon 3D OCT-1 Maestro (3D OCT-1 Maestro, Topcon, Tokyo) spectral domain optic coherence tomography system with an anterior segment lens to obtain corneal epithelial thickness values. The instrument operates fully automatically, captures 50,000 axial scans per second, and has an in-depth resolution of 6 μm. The device provides the corneal image with a 6×6 mm automated segmentation OCT scan. Maximal epithelial thickness measurements were taken at the thickest epithelial point over the lesions in both groups with using images obtained from the device [7]. Incisional or excisional biopsies were performed under local anesthesia in the operating room. Regarding anatomical position, all excisional biopsy samples were noted. The ocular pathology department of the hospital received all biopsy samples for histologic analysis and evaluation.

Statistical analysis

Data analysis were performed using IBM SPSS 25.0 (IBM Corp., Armonk, NY) and MedCalc 15.8 (MedCalc Software bvba, Ostend, Belgium) statistical package programs. The Chi-square (χ^2^) test was used to compare qualitative data as well as descriptive statistical methods (frequency, percentage, mean, standard deviation, median, and min-max). The suitability of the data to the normal distribution was evaluated using the Kolmogorov-Smirnov test, skewness-kurtosis, and graphical methods (histogram, Q-Q plot, stem-and-leaf plot, and box plot). Independent samples t-test (t-test for independent groups) was used in the evaluation of the quantitative data showing normal distribution. The receiver operating characteristic (ROC) curve method was used to determine the distinctiveness of the variables, the binary logistic regression test was used to determine the risk ratios, and Kaplan-Meier, Log Rank, Breslow and Tarone-Ware tests were used for survival and survival analysis. The statistical significance level was accepted as a=0.05.

## Results

Ocular surface squamous neoplasia was histopathologically identified in 21 patients, and a pterygium-like tumor on the conjunctiva was identified in 19 patients. The histopathological findings were all consistent with the clinical examination-based diagnosis. Demographic data and the comparisons for the two groups are presented in Table 1. The average age of patients with OSSN was 51.6 ± 17.8 and 49.5 ± 12.2 with pterygium. Of the patients with OSSN, 57.1% had lesions at the nasal and 28.6% at the temporal quadrants.

**Table 1:** Demographic properties and lesion characteristics in patients with OSSN and pterygium. ^a^Chi-square test (n (%)), ^b^Independent samples t-test (mean ± SD). OSSN: ocular surface squamous neoplasia.

		Pterygium (n=19)	OSSN (n=21)	P
Gender	Female	10 (52.6%)	8 (38.1%)	0.545^a^
	Male	9 (47.4%)	13 (61.9%)	
Age		49.5 ± 12.2	51.6 ± 17.8	0.671^b^
Involved eye	Right	13 (68.4%)	13 (61.9%)	0.921^a^
	Left	6 (31.6%)	8 (38.1%)	
Quadrant	Nasal	18 (94.7%)	12 (57.1%)	-
	Temporal	1 (5.3%)	6 (28.6%)	
	Inferior	0 (0.0%)	1 (4.8%)	
	More than 2	0 (0.0%)	1 (4.8%)	
	Superior	0 (0.0%)	1 (4.8%)	
Epithelial thickness		80.7 ± 43.4	295.3 ± 111.3	0.000^b^

The mean epithelial thickness values between the OSSN and pterygium patients were compared, and the maximal epithelial thickness values were measured through the thickest point in each lesion using AS-OCT. The mean epithelial thickness was found to be 295.3 ± 111.3 µm in OSSN patients and 80.7 ± 43.4 µm in pterygium patients (P<0.005). The receiver operating characteristic (ROC) curve showed almost no overlap between the two groups, with an area under the curve of 98% p<0.001, 95% CI: 0.876-1.000. The ROC curve analysis revealed a cut-off value of 97 µm for the differential diagnosis of OSSN from pterygium, with a sensitivity of 100% and specificity of 94.7%.

**Figure 1:**
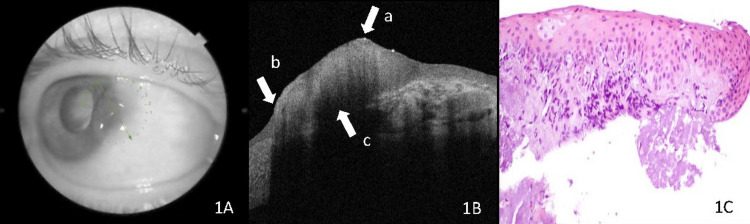
Optic coherence tomography (OCT) image of ocular surface squamous neoplasia (OSSN). (A) OCT photographs demonstrating OSSN, the green arrow represents the direction and location of the anterior segment OCT scans. (B) OCT images of the lesion demonstrating a thickened hyperreflective epithelium (arrow a). Abrupt transition between abnormal and normal epithelium (arrow b). There is a line of cleavage between the tumor and the surrounding tissue (arrow c). (C) Histopathological specimens from these lesions demonstrate a mucosal epithelium with dysplasia consisting of epithelial cells with hyperchromatic nuclei (stain, hematoxylin-eosin; ×20).

AS-OCT demonstrated the OSSN lesions as thickened, hyperreflective epithelium with an abrupt transition between the normal and affected epithelium. A plane of cleavage between the lesion and underlying normal tissue was noted in most cases (Figures 1A-1B). Histopathological examination of the OSSN patients revealed acanthotic conjunctival and/or corneal epithelium with partial or full-thickness dysplastic findings without invasion of stromal tissue (Figure 1C).

**Figure 2:**
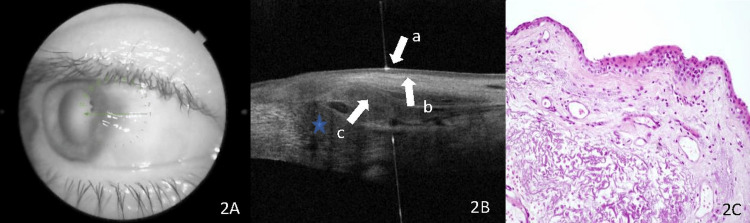
OCT image of pterygium. (A) Optic coherence tomography (OCT) showing conjunctival and corneal lesions that are compatible with a pterygium. The green arrow represents the direction and location of the anterior segment OCT scan. (B) OCT image demonstrates the line of cleavage (arrow c) divides thick subepithelial hyperreflective lesions (arrow a) from the overlying epithelium. Blood vessels can be seen as areas of hyporeflectivity in the subepithelial tissue of the pterygium tissue (blue star). The lesion's epithelium is thin or in normal thickness, and it only exhibits moderate hyperreflectivity (arrow b). (C) Histopathologic specimens from these lesions revealing signs of epithelial atrophy, mild calcification of the stroma, minimal vascular proliferation, and diffuse elastic degeneration (stain, hematoxylin-eosin; ×20).

Histopathological evaluation and AS-OCT imaging in pterygium patients showed correlated results with each other. A thin epithelium with underlying subepithelial hyperreflective tissue was observed in AS-OCT images of all the patients with pterygium (Figures 2A-2B). A normal conjunctival epithelium without acanthosis was demonstrated in histological specimens (Figure 2C).

## Discussion

The spectrum of OSSN includes a wide variety of conditions characterized by the aberrant development of dysplastic squamous epithelial cells on the ocular surface. Patients typically have a gelatinous or plaque-like interpalpebral conjunctival gray or whitish lesion when the lesions appear [8]. Limbus is the area where the most actively mitotic cells reside [9]. Corneal involvement is the result of the spread of abnormal epithelium from the adjacent limbus. OSSN has been identified using OCT as an in vivo diagnosis tool. When compared to other conjunctival tumors like pterygium, the distinctive characteristics of OSSN, such as hyperreflectivity, thickened epithelium, and abrupt transition from normal to abnormal tissue, serve to identify it.

OCT has been employed as an in vivo diagnostic modality in the detection of OSSN. Distinctive features of OSSN, such as hyperreflectivity and thickened epithelium, and an abrupt transition from normal to abnormal tissue detected on AS-OCT help to differentiate it from other conjunctival lesions such as pterygium. There are reports in the literature where invasive squamous cell carcinoma of the conjunctiva is clinically misdiagnosed as pterygium [10,11]. In this study, we examined the correlation of AS-OCT images and pathological evaluation in the diagnosis of OSSN and pterygium and found a cut-off value for epithelial thickness to help clinicians in distinguishing between OSSN and pterygium.

High (5-10 μm) and ultra-high (<5) resolution AS-OCT devices made epithelium resolution from underlying tissue layers possible, which in turn caused more precise diagnosis of OSSN by means of noninvasive diagnostic methods [12]. Kieval et al. [13] demonstrated that the mean epithelial thickness was higher in OSSN patients than in pterygium patients by using ultra-high resolution AS-OCT (P<0.001). In the same study, they found a cut-off value of 142 µm to reach the OSSN diagnosis with a sensitivity of 94% and a specificity of 100%. High-resolution OCT (HR-OCT) can also attain an axial resolution of less than 5 μm, which elucidates enough detail to allow reliable assessment of OSSN. Thus, Nanji et al. [14] employed high-resolution AS-OCT to reach 120 μm as a cut-off value for differentiating between OSSN and pterygia, with a sensitivity and specificity of 100%. Comparable to the literature results, we found that the mean epithelial thickness values were significantly different between the two groups; the OSSN group being higher​​ (p<0.05). In our study, we also found that the 97 µm epithelial thickness value in AS-OCT helps to differentiate OSSN and pterygium with a 100% sensitivity and 94.7% specificity. We believe that our study provides additional data to the literature on the use of AS-OCT in the differential diagnosis of OSSN patients. Lesions with epithelial thickness values above 97 µm should be carefully evaluated.

Although the histopathological examination is still the gold standard for the diagnosis of OSSN, the outcomes of earlier trials using AS-OCT were impressive [5,6,13]. AS-OCT allows clinicians to detect the presence of morphologically suspicious elements in the lesions in a minimally invasive manner. The number of unnecessary biopsies, surgical excision, cryotherapy, and topical chemotherapeutic agent use might be diminished in some patients with the use of AS-OCT. AS-OCT can also assist clinicians in the early detection of recurrences, and it can help to determine the margin of excision if a surgical excision is planned in the recurrences. Impression cytologic analysis and confocal microscopy have also been used in the clinics for the diagnosis of OSSN. However, the sensitivity of the impression cytologic analysis seems to be low. Nolan et al. [15] found that only 77-80% of the positive cytologic specimens were confirmed by histopathologic examination. Confocal microscopy was also reported as an effective device to differentiate between normal and abnormal epithelium [3,4,15]. Still, AS-OCT seems to be superior to confocal microscopy because of its rapid image capture property, being a noncontact method and providing a cross-sectional view of the tissue. In our study, AS-OCT scans revealed compatible results with the histopathologic evaluation. AS-OCT scan results effectively showed that OSSN lesions consisted of a thickened abnormal epithelium, which is different from a pterygium that shows normal epithelial and occasionally subepithelial actinic changes. It is a setback for AS-OCT devices that tumor invasion or the grade cannot be detected. Plus, leukoplakic or hyperreflective lesions can prevent the visualization of the underlying tissue. Nevertheless, epithelial thickness measurements still can be obtained.

Retrospective design is the main limitation of our study. At our institution, AS-OCT is employed for regular monitoring of the ocular surface of patients diagnosed with, and treated for, OSSN and pterygium. Therefore, we retrospectively evaluated the accumulated data in our clinic. However, a prospective study focusing on the clinical and AS-OCT imaging-based resolution of the lesions after medical treatment with chemotherapeutic agents is also planned.

## Conclusions

In conclusion, AS-OCT is a noninvasive technology that may be used in the differential diagnosis and monitoring of OSSN patients. The results of AS-OCT are consistent with the histopathologic samples. This indicates that AS-OCT could be reliable in clinical use. It can also be used to effectively monitor for recurrences following surgical excision or medical treatment. In addition, the cut-off value obtained by ROC analysis in our study may provide convenience as a reference value in the measurement of lesions. It could be possible to follow up with individuals who don't want surgery right away and those with suspicious lesions that are benign in appearance. Lesion-specific findings will be better recorded with the increased usage of higher-resolution OCT devices in anterior segment lesions. Hence, the clinical characteristics and documented findings of the conjunctival lesions will aid doctors in making surgical decisions in clinics.
